# Subgroup and outlier detection analysis

**DOI:** 10.1186/1471-2105-14-S17-A2

**Published:** 2013-10-22

**Authors:** Gang Wu, Iwona Pawlikowska, Tanja Gruber, James Downing, Jinghui Zhang, Stan Pounds

**Affiliations:** 1 Department of Computational Biology, St. Jude Children’s Research Hospital, Memphis, TN 38105, USA; 2 Department of Biostatistics, St. Jude Children’s Research Hospital, Memphis, TN 38105, USA; 3 Department of Oncology, St. Jude Children’s Research Hospital, Memphis, TN 38105, USA; 4 Department of Pathology, St. Jude Children’s Research Hospital, Memphis, TN 38105, USA

## Background

High-dimensional biological data presents the opportunity to discover novel forms of biological heterogeneity, such as overexpression or suppression of expression of a particular gene in a subset of a cohort. This novel biological heterogeneity appears in the data as outliers or distinct subgroups. Here, we describe and evaluate three procedures for subgroup and outlier detection analysis (SODA): a leave-one-out (LOO) procedure that is widely used for outlier detection in the bioinformatics literature, the least median squares (LMS) procedure from the statistics literature, and the dip test (DT) from the statistics literature. We also propose and evaluate the max spacing test (MST) as a novel SODA method.

## Results

In simulation studies, we found that LMS, DT, and MST are each the best method in specific settings. In an example analysis, we found that LMS and MST effectively identified confirmed fusion genes as outliers and DT and MST effectively identified genes that distinguish between two confirmed subtypes of pediatric acute megakaryoblastic leukemia. We conclude that LMS, DT, and MST are robust and complimentary methods for SODA.

